# Lipid absorption and overall intestinal lymphatic transport are impaired following partial small bowel resection in mice

**DOI:** 10.1038/s41598-022-15848-6

**Published:** 2022-07-07

**Authors:** Emily J. Onufer, Rafael S. Czepielewski, Yong-Hyun Han, Cathleen M. Courtney, Stephanie Sutton, Anne Sescleifer, Gwendalyn J. Randolph, Brad W. Warner

**Affiliations:** 1 Division of Pediatric Surgery, Department of Surgery, St. Louis Children’s Hospital, Washington University in St. Louis School of Medicine, St. Louis, MO USA; 2 Department of Pathology and Immunology, Washington University in St. Louis, St. Louis, MO USA; 3 Laboratory of Pathology and Physiology, College of Pharmacy, Kangwon National University, Chuncheon, 24341 South Korea; 4 St. Louis University School of Medicine, St. Louis, MO USA

**Keywords:** Diseases, Gastroenterology

## Abstract

Short bowel syndrome (SBS) is associated with diminished levels of serum fats caused by unknown mechanisms. We have shown that mesenteric lymphatics remodel to a more primitive state one week after small bowel resection (SBR); therefore, this study focuses on the effect of chronic lymphatic remodeling and magnitude of resection on intestinal lipid uptake and transport. C57BL6 and Prox1 creER-Rosa26^LSL^TdTomato (lymphatic reporter) mice underwent 50% or 75% proximal SBR or sham operations. Functional transport of lipids and fecal fat content was measured and lymphatic vasculature was compared via imaging. There was a significant reduction in functional transport of cholesterol and triglyceride after SBR with increasing loss of bowel, mirrored by a progressive increase in fecal fat content. We also describe significant morphological changes in the lymphatic vasculature in both the lamina propria and mesentery. Intestinal lymphatic drainage assay in vivo demonstrated a marked reduction of systemic absorption after resection. Intestinal lymphatic vessels significantly remodel in the setting of chronic SBS. This remodeling may account at least in part for impaired intestinal uptake and transport of fat via the compromised lymphatic architecture. We believe that these changes may contribute to the development of intestinal failure associated liver disease (IFALD), a major morbidity in patients with SBS.

## Introduction

Short bowel syndrome (SBS) is a morbid clinical condition resulting from the massive loss of small intestine due to surgical resection. While such resections may be clinically necessary in settings as diverse as necrotizing enterocolitis, trauma, or inflammatory bowel disease, these radical surgeries have their own potentially adverse consequences. The remaining bowel may be unable to absorb and/or digest substantive nutrients for maintenance and growth^1–3^. This failure can arise despite the remnant bowel undergoing a compensatory adaptation response to increase function via expansion of intestinal surface area. Consequently, many SBS patients still require parenteral nutrition for additional supplementation^4^.

One class of these important macronutrient in the context of SBS is dietary fat. While fatty acids and cholesterol can be synthesized de novo, the uptake of these lipids occurs concomitant with essential fatty acids and lipophilic vitamins that are not synthesized and must be acquired from the diet. Lipid absorption relies on the generation of lipoproteins called chylomicrons that are assembled and secreted by intestinal epithelial cells in the proximal small bowel and subsequently transported into intestine-draining lymphatic vessels^5^. Bile acids, derived from hepatic cholesterol metabolism, reach the proximal gut through the bile duct, where they facilitate emulsification of dietary fats that allows epithelial uptake and repackaging of ingested lipids into chylomicrons. In turn, re-absorption of biliary bile acids occurs in the distal small intestine^6^. Diet-derived plant sterols, such as campesterol, enter the body via chylomicrons and thereby serve as a surrogate marker for dietary lipid absorption^7–10^. When lipid absorption is impaired, the ratio of plasma lathesterol (a surrogate for endogenous cholesterol synthesis) to campesterol is frequently increased^11–13^. Indeed, there is a significant reduction in campesterol in the plasma of SBS patients after withdrawal from parenteral nutrition^14^, raising the possibility that these patients may fail to absorb lipid nutrients from the diet normally. While diminished intestinal epithelial cell populations is thought to account for malabsorption of lipids observed in patients with SBS, other possible contributing factors like failed transport of cargo through the lymphatic vasculature has not been assessed. Addressing these possibilities is critical to optimizing and improving clinical management of SBS patients.

We had previously reported extensive structural remodeling of mesenteric lymphatic vessels draining the intestine within a week after experimental small bowel resection (SBR) in mice^15^. However, it remained unclear whether this remodeling persisted beyond an initial recovery from surgery and whether lymphatic transport was functionally impacted by the remodeling. Here, we reveal that lymphatic cargo, including but not limited to dietary lipids, fail to traffic from the gut to the host circulation or draining lymph nodes. These findings highlight the need to consider diminished lymphatic transport in therapeutic management of SBS.

## Results

### Malabsorption of cholesterol and triglycerides after SBR

**Figure 1:**
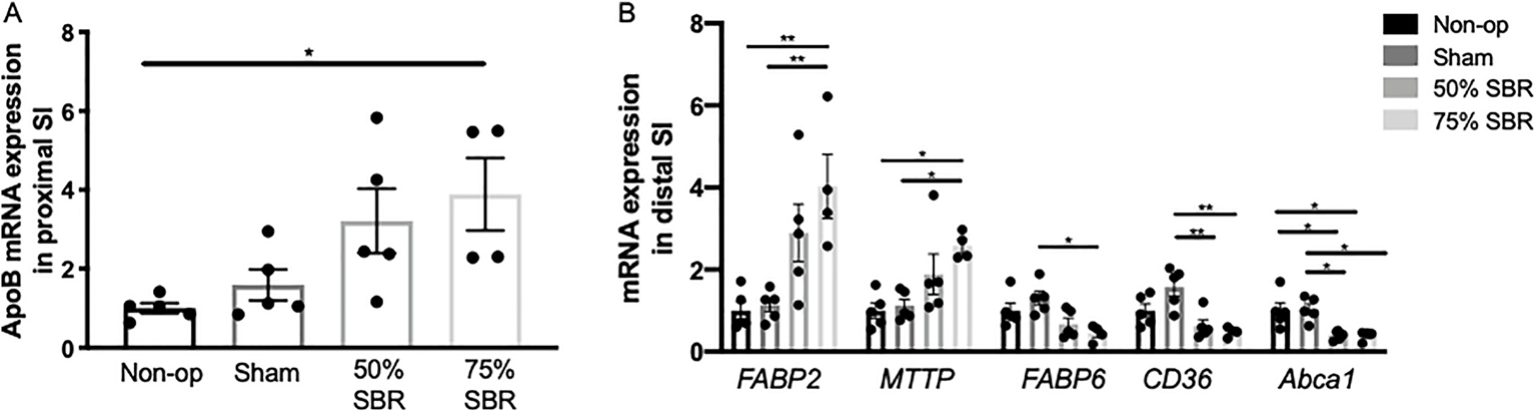
(**A**) mRNA expression levels of intestinal fatty acid transporters in the (**B**) proximal and (**B**) distal small intestine (SI) in non-op (*n* = 5), sham (*n* = 5), 50% SBR (*n* = 5), and 75% SBR (*n* = 4) mice. *p < 0.05, **p < 0.005.

At an early time point after resection, postoperative day 7, the distal intestine reprograms to a more proximal identity with an upregulation of genes associated with lipid handling and metabolism^16^. In agreement with this reprogramming, we observed a progressive increase in expression of *ApoB* in the proximal intestine and *MTTP* in the remaining distal intestinal segment, which regulate the assembly of chylomicrons, in the resected mice (Fig. 1A, B; ANOVA *p* < 0.05 and *p* < 0.01, respectively). As we have previously shown in single-cell analysis at early time points after resection, there was an increase in the intestinal lipid-sensing gene *FABP2* in the distal intestine (Fig. 1B; ANOVA *p* < 0.005)^16^. Simultaneously, in the distal intestine, there was a decrease in the long-chain fatty acid transporter gene, *CD36,* the cholesterol transporter gene, *Abca1*, and the bile acid binding gene, *FABP6*, expression in the distal intestine with increasing loss of bowel (Fig. 1B; ANOVA *p* < 0.005, *p* < 0.005, and *p* < 0.05, respectively). These data show that the intestine reprograms to promote lipid absorption and chylomicron assembly, leading us to test whether fat absorption normalized in concert with this reprogramming.

**Figure 2:**
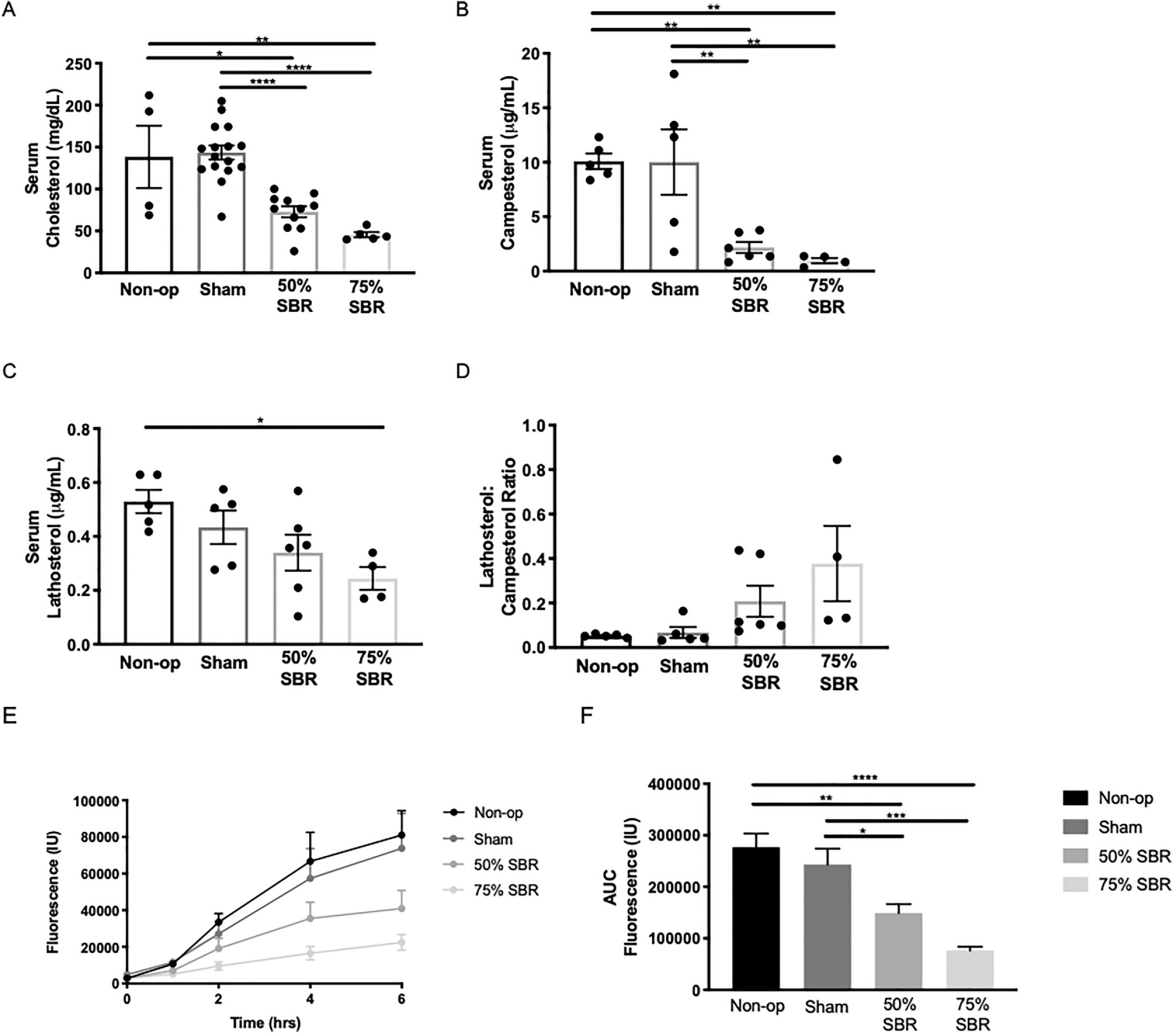
Effects of SBR on cholesterol and its derivatives. (**A**) Serum cholesterol levels in non-op (*n* = 4), sham (*n* = 16), 50% SBR (*n* = 11), and 75% SBR (*n* = 5) mice. (**B**) Serum campesterol levels, (**C**) lathosterol levels, and (**D**) lathosterol:campesterol ratios in non-op (*n* = 5), sham (*n* = 5), 50% SBR (*n* = 6), 75% SBR (*n* = 4) mice. Exogenous absorption of TopFluor-cholesterol assessed via fluorescence over (**E**) time course and as (**F**) area under the curve in non-op (*n* = 5), sham (*n* = 6), 50% SBR (*n* = 6), and 75% SBR (*n* = 5) mice. *p < 0.05, **p < 0.005, ***p < 0.001, ****p < 0.0001.

Despite the favorable programming of genes to orient toward fat absorption, with increasing loss of bowel, total serum cholesterol decreased with 75% resected mice having a 67% decrease compared to non-operated controls (Fig. 2A; ANOVA *p* < 0.0001). Serum campesterol, a marker of dietary cholesterol absorption, followed this same trend of decreased concentrations with increased extent of bowel resection (Fig. 2B; ANOVA *p* < 0.001). Lathosterol, an intermediate in cholesterol synthesis, also decreased in the serum as proportion of intestine resected increased (Fig. 2C; ANOVA *p* < 0.05). However, as the lathosterol to campesterol ratio increased with the magnitude of resection (Fig. 2D), the extent of malabsorption appears greater than the decrease in cholesterol synthesis. To directly assess absorption of exogenous cholesterol, we performed a time-course of serum fluorescence following oral delivery of the fluorescently labeled cholesterol mimetic TOPFLUOR cholesterol. With increasing loss of bowel, there was a decreasing amount of TOPFLUOR cholesterol absorbed (Fig. 2E, F; ANOVA *p* < 0.0001).

**Figure 3:**
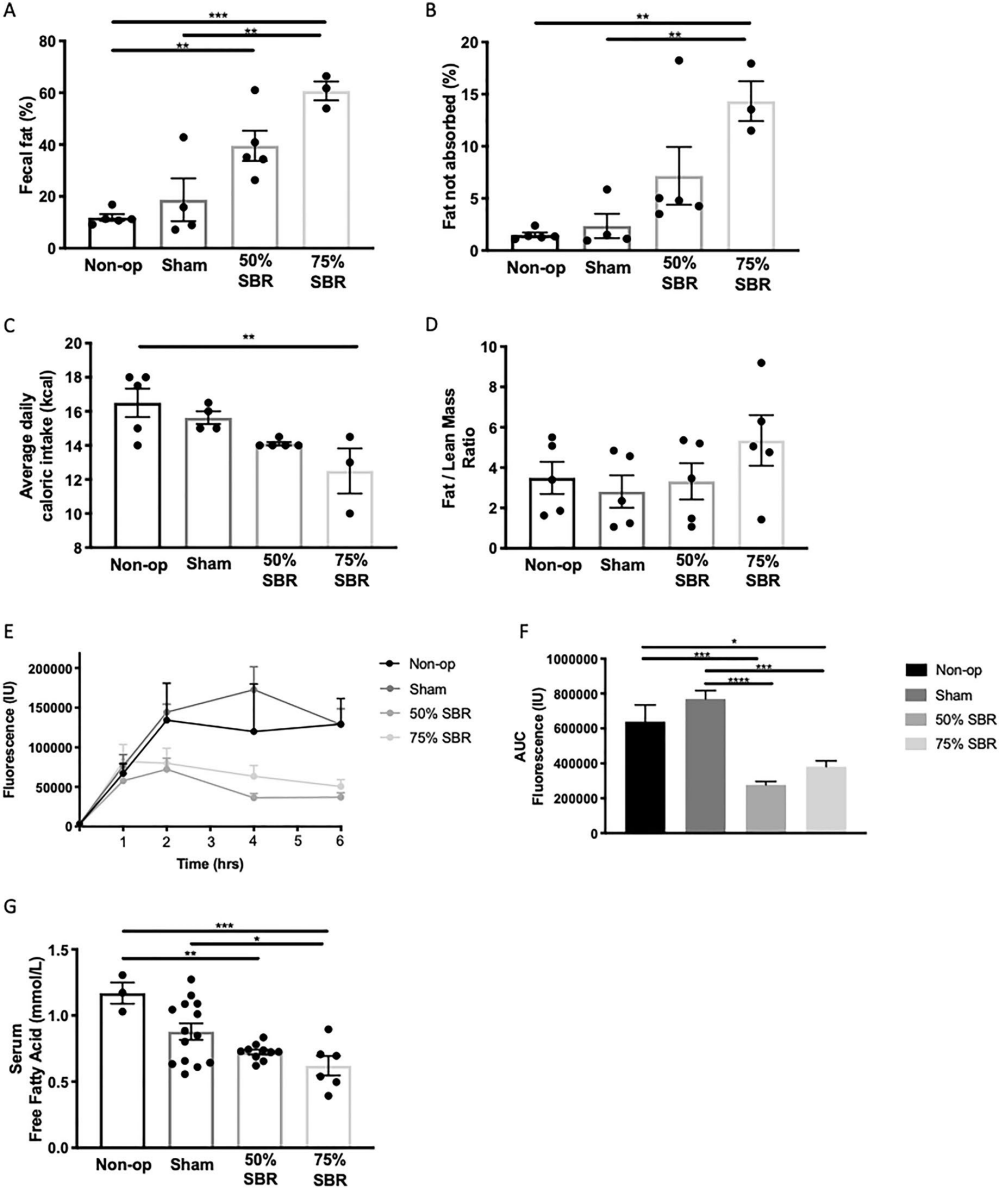
(**A**) Fecal fat, (**B**) % fat unabsorbed, and (**C**) average daily caloric intake over two days at approximately one year after resection in non-op (*n* = 5), sham (*n* = 4), 50% SBR (*n* = 5), and 75% SBR (*n* = 3) mice. (**D**) Fat to lean mass ratio at approximately one year after resection in non-op, sham, 50% SBR, and 75% SBR mice (*n* = 5 for all groups). (**E**) Kinetic C16-Bodipy-labeled olive oil fluorescence and (**F**) area under the curve in non-op (*n* = 5), sham (*n* = 7), 50% SBR (*n* = 6), and 75% SBR (*n* = 5) mice. (**G**) Serum free fatty acid levels non-op (*n* = 3), sham (*n* = 14), 50% SBR (*n* = 9), and 75% SBR (*n* = 6) mice. *p < 0.05, **p < 0.01, ***p < 0.001.

These data pointed to failed absorption of fat, leading to seek other ways to confirm this observation. Failed absorption of dietary fat would be expected to result from increased fat content in feces. At one year after SBR, with increasing loss of bowel after resection, fecal fat concomitantly increased (Fig. 3A, B; ANOVA *p* < 0.0005), a feature that held true when the data were normalized to average caloric intake (Fig. 3C; ANOVA *p* < 0.05). There was no difference in fat to lean mass ratios across the groups (Fig. 3D). To assess chylomicron transport via lymphatics, we performed a functional assay of lipid absorption using C16-Bodipy-labeled olive oil (C16-Bodipy is a neutral lipid that binds to olive oil upon mixture). This showed a decrease in chylomicron absorption with a lipid load over time in the resected mice (Fig. 3E, F; ANOVA *p* < 0.0001). Serum free fatty acids at 12–15 weeks after resection also followed this same trend (Fig. 3G; ANOVA *p* = 0.0007).

**Figure 4:**
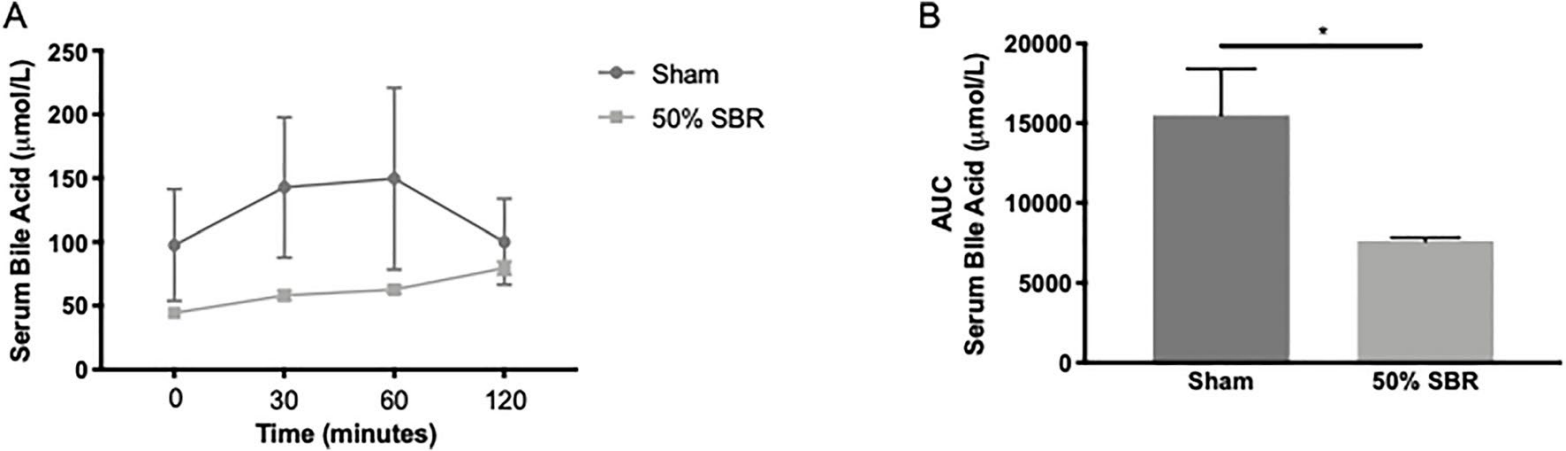
(**A**) Kinetic bile acid absorption and (**B**) area under the curve following an oral lipid bolus in sham (*n* = 5) and 50% SBR (*n* = 5) mice. *p < 0.05.

These data indicate that failed intestinal lipid absorption is present in mice long after surgical resection. Overall, we conclude that fat malabsorption exists as a chronic consequence of SBR. SBR promoted a 51% reduction in serum bile acids after an oral fat load, which may result in reduced secretion of bile acids in the intestinal lumen to promote lipid uptake (Fig. 4A, B; *p* < 0.05). However, as an additional mechanism, there may also be a role for lymphatic remodeling, which we next set out to explore. The data so far reveal reduced food intake of mice with 75% SBR and higher fecal fat. Yet, absorption defects were similar between mice with 50% versus 75% SBR. Thus, to proceed with fewer confounding factors, we continued our studies focusing on the lymphatic vasculature in mice receiving 50% SBR.

### Intestinal mucosal lymphatic capillaries change after chronic SBR

**Figure 5:**
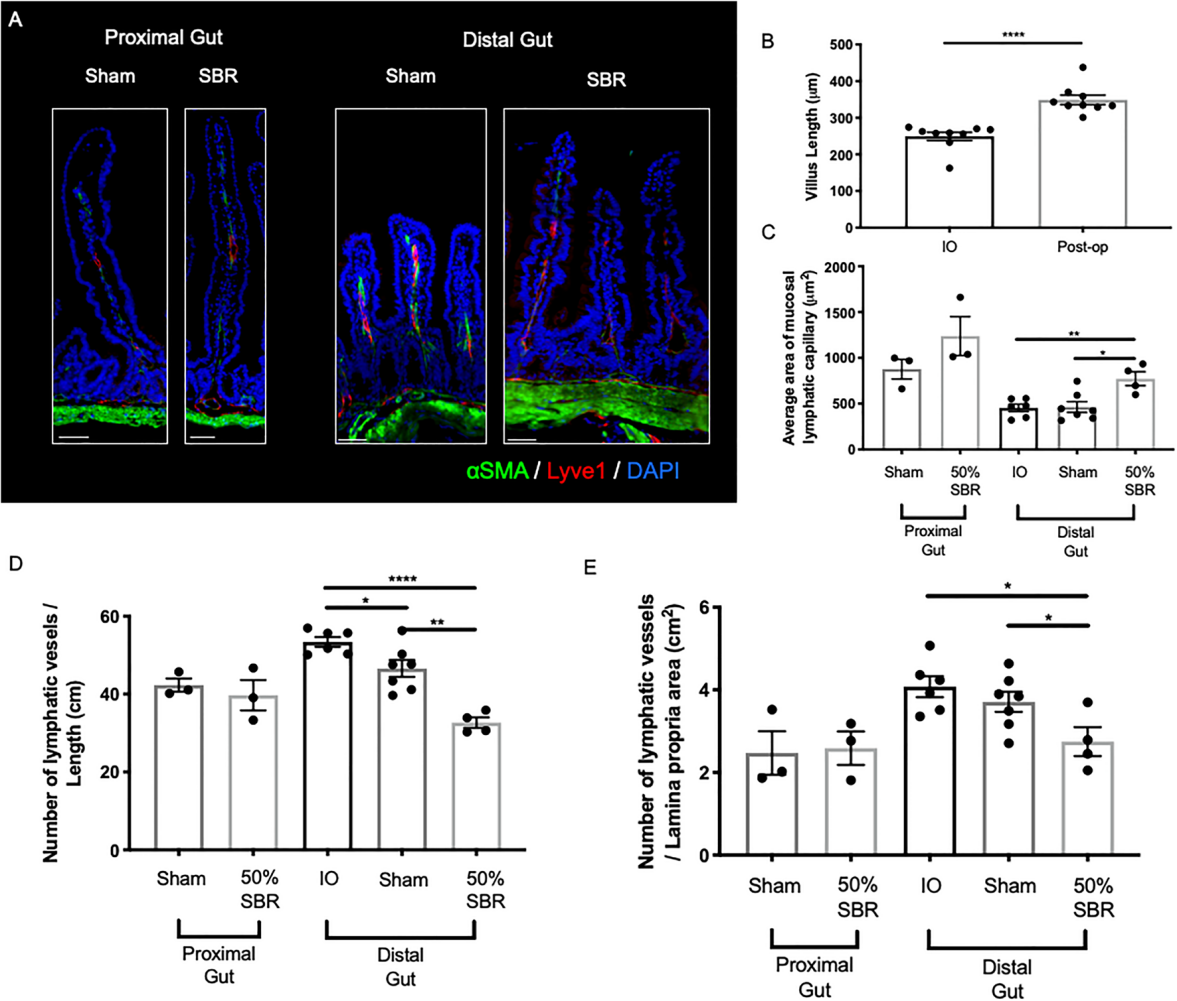
Chronic mucosal lymphatic capillary changes in SBR vs. sham mice. (**A**) Representative immunofluorescence images of proximal (left side) and distal (right side) sham and 50% SBR intestinal tissue sections stained for smooth muscle (alpha-smooth muscle actin, αSMA, green), lymphatics (LYVE-1, red), and nuclei (DAPI, blue) on post-operative week 15 and 12, respectively. Lyve1 + vessels were considered mucosal lymphatics when present in the lamina propria (defined as the space between the basal membrane and muscularis mucosa in αSMA, green). Scale bar 30 μm. (**B**) Villus height increased by an average of 40% in 50% resected mice (*n* = 9 for IO and Post-op), assuring adaptation. (**C**) Average area of mucosal lymphatic capillaries in proximal (sham *n* = 3, 50% SBR *n* = 3) and distal (intraoperative *n* = 6, sham *n* = 7, 50% SBR *n* = 4) intestine. The number of mucosal lymphatic capillaries (Lyve1 + vessels as in A) per (**D**) length and (**E**) total area of the lamina propria in proximal and distal intestinal tissue. *p < 0.05, **p < 0.005, ****p < 0.0001.

We have previously shown dilation of the upstream mucosal lymphatic capillaries in the remnant distal bowel seven days after resection^15^, but it was unclear if over time the remodeled lymphatics returned to their baseline arrangement or remained remodeled. We thus extended the period that we assessed the lymphatic vasculature to 3–4 months following surgical resection. In cross-sectional images of the small intestine (Fig. 5A), we observed the previously documented 40% increase in villus height by post-operative week 12–15 (Fig. 5B; *p* < 0.0001), serving as a positive control for adaptive remodeling. Lymphatic capillary luminal area in the lamina propria was significantly increased in the distal intestine by 66% and 70% in resected mice compared to sham and intraoperative controls, respectively; there was also a pattern of increasing lymphatic area in the proximal intestine (Fig. 5C; *p* < 0.05, *p* < 0.005). Image analysis for the number lymphatic capillary vessels by both the total length and the total lamina propria area showed no change in the proximal intestine but significantly decreased in the 50% resected mice compared to both intraoperative and sham controls in the distal intestine (Fig. 5D, E).

### Mesenteric collecting lymphatic vessel changes after chronic SBR

**Figure 6:**
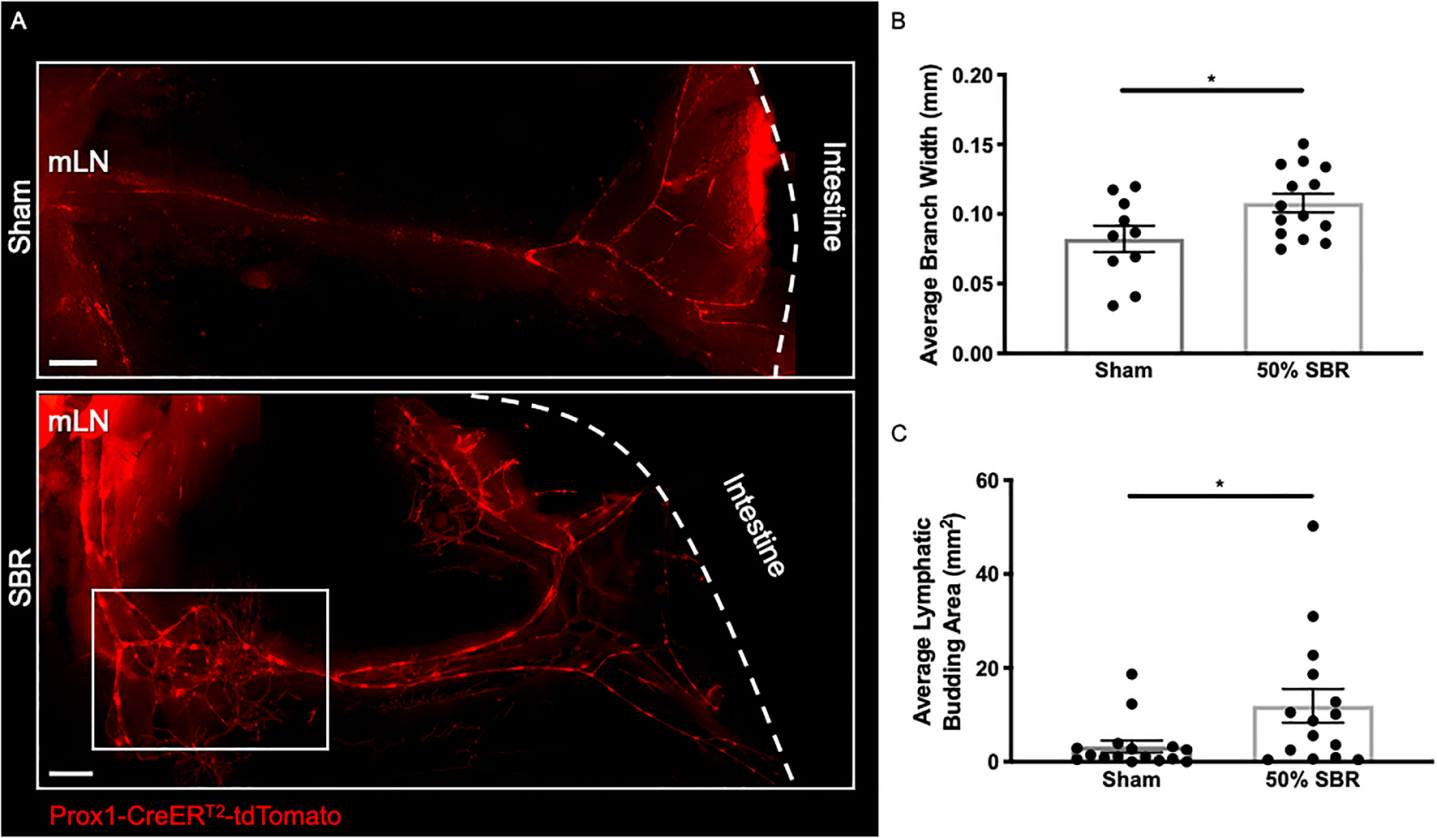
Effect of SBR on mesenteric lymphatic vessels at post-operative week 13. (**A**) Whole mount fluorescence stereoscope image of collecting lymphatic vessels (Prox1-tdTomato +) (red) in sham and 50% SBR mice in regions in the ileum-draining mesentery distal to the anastomosis. Example of budding area is outlined by white box; mesenteric lymph node (mLN) is designated to the left and intestine to the right (white dashed line). Scale bar 1000 μm. (**B**) Average width of lymphatic branches and (**C**) average lymphatic budding area draining the bowel distal to the anastomosis in sham (*n* = 10 branches in 4 mice) and 50% SBR (*n* = 14 branches in 4 mice). *p < 0.05.

In addition to the alterations observed in the distal small intestine lymphatic mucosal capillary network, we examined the effect of resection on the mesenteric lymphatic vessels. At postoperative week 13, collecting lymphatic vessels present in the mesentery and emerging from the intestinal wall distal to the anastomosis still showed the substantially altered morphology following 50% SBR versus sham control mice (Fig. 6A) that we had earlier observed at day 7^15^. The resected mice had an average 31% increase in mesenteric branch width compared to sham controls (Fig. 6B; *p* < 0.05). Furthermore, resected mice had an average 31% increase in lymphatic budding area into the mesenteric sheath compared to sham controls (Fig. 6C; *p* < 0.05).

### Compromise of lymphatic flow after intestinal resection

**Figure 7:**
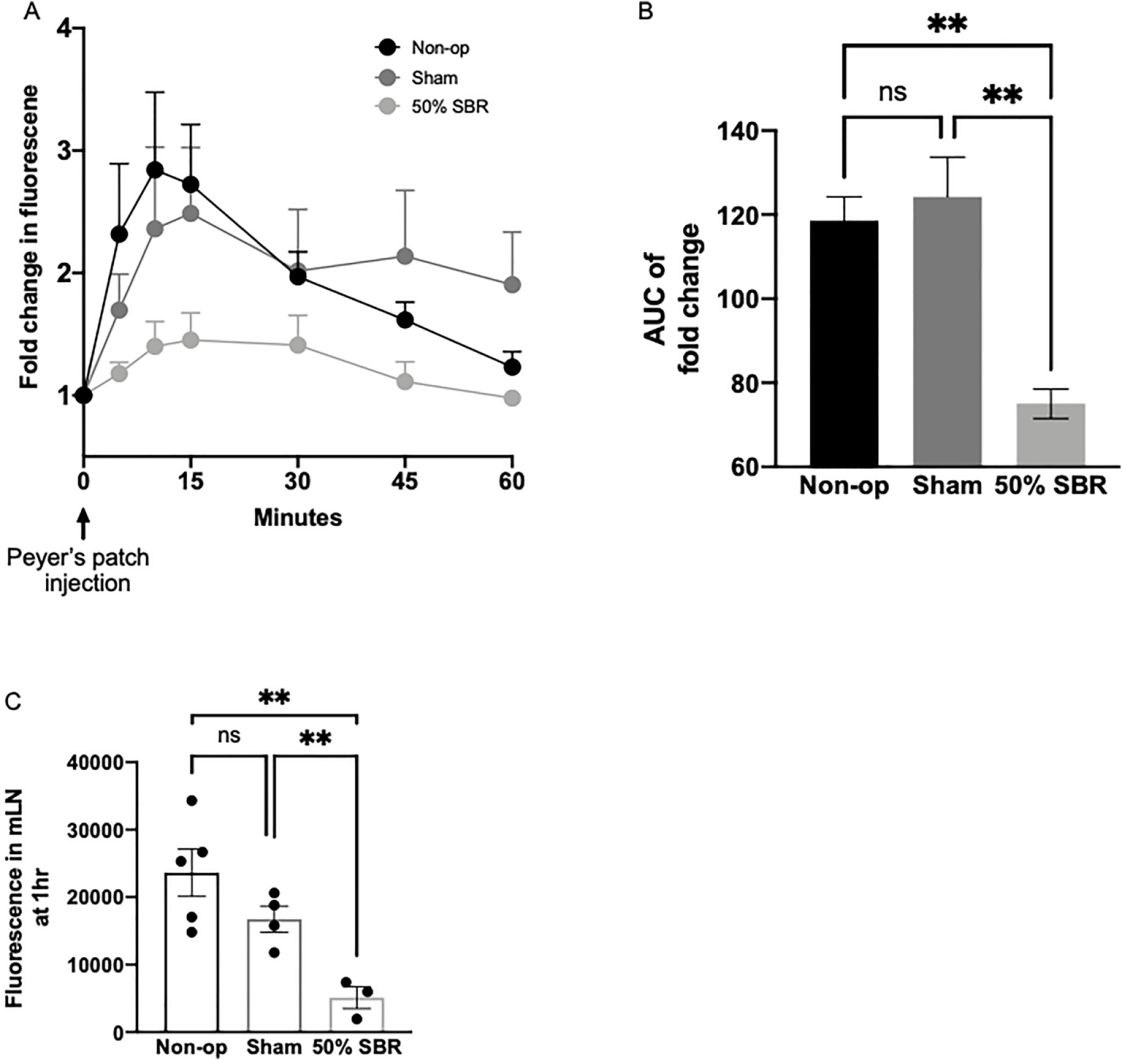
(**A**) Kinetic measurement of lymphatic flow from Peyer’s patch injection to systemic blood by fluorescence, presented in fold change from pre-injection baseline, in non-op (*n* = 8), sham (*n* = 6), and 50% SBR (*n* = 4) mice, with resultant (**B**) area under the curve. (C) Measurement of fluorescence in the mesenteric lymph node (mLN) at one hour in non-op (*n* = 3), sham (*n* = 4), and 50% SBR (*n* = 3) mice after Peyer’s patch injection. **p < 0.005.

The findings above collectively revealed that lymphatic remodeling persisted chronically after SBR. We thus wondered if lymph transport might be altered at these time points. To test the intestinal lymphatic drainage function in SBR, we performed a standardized micro injection of a fluorescent marker into Peyer’s patches in vivo with timed measurements via serum fluorescence. This experiment tests that capacity of the lymphatic system to transport soluble cargo from the intestine in the lymph, of which chylomicrons are a major component. Gut that drains to the downstream mesenteric lymphatic network showed compromised flow in resected mice compared to controls (Fig. 7A, B; ANOVA *p* < 0.05). Accumulation of the tracer in the draining lymph node was 78% and 70% reduction in mice receiving surgical removal of 50% of the small bowel compared to nonoperative and sham controls, respectively (Fig. 7C; *p* < 0.005). These data reveal that marked impairment of lymphatic transport accompanies the altered morphology and may account at least in part for the failed absorption of lipid nutrients.

## Discussion

Here, we show by analysis of fecal fat content and by tracing the fate of orally delivered fluorescent cholesterol analogue that dietary lipid absorption is impaired after experimental SBR many weeks beyond the point of surgical resection of the small bowel. Furthermore, we found a significant reduction in the blood cholesterol metabolite campesterol in our mice. This reduction resembles a similar trend in the plasma of SBS patients that have been studied after withdrawal from parenteral nutrition^14^. Because campesterol arrives to plasma via absorption from the gut, the finding further supported the conclusion of impaired absorption or transport, although reduction in diet-derived campesterol might in part be due to partially reduced food intake in mice receiving 75% SBR. All nutrient absorption is compromised in SBS patients due to the loss of absorptive surface area, lipid absorption is considered to be the most vulnerable, in part due to compromise of the enterohepatic circulation, decreased bile acid pool, and decreased pancreatic lipase secretion^17–19^.

After establishing evidence for failed lipid absorption persisting after SBR in mice, the question became what mechanisms are causal. Failed transport of fats may indicate impaired duodenal and jejunal lymph transport, as lipid absorption is most prominent in these sites. Alternatively or additionally, impaired lipid transport might point toward reduced capacity of the gut to take up or package fats. The bile acid pool was reduced, and indeed may contribute importantly to reduced lipid uptake. In addition, our findings suggest that the lymphatic vasculature was also impaired. Besides morphological changes in the lymphatic vasculature after SBR, fluorescent dextran tracer deposited in the submucosa-draining lymphatic network of the intestine confirmed significantly impaired lymph flow using an approach without confounding possible changes in absorption.

One week after resection, early lymphatic remodeling in the intestinal mucosal lymphatics is characterized by reduced mucosal lymphatic capillary vessel area^15^. Interestingly, after a prolonged period of resection, we have shown that lymphatic capillary area actually increases compared to both intraoperative and sham controls in the distal intestine. We believe that is likely secondary obstruction of flow in the mesentery causing upstream dilation in the intestinal mucosa. We also witnessed this in the mesenteric collecting vessels as the branch width was also dilated after resection. Similar to prior observations seen at POD7, resected mice also had an increase in lymphatic budding area into its mesenteric sheath compared to controls. This budding is normally observed during early development in mice^20,21^.

In addition to possibly direct effects on lipid absorption along the epithelium, could failed lymphatic transport impact lipid absorption such that lipid remained luminal in the intestine and ultimately appeared in the feces? While the idea that altered lymphatic function might impact absorption across the epithelium may seem counterintuitive, there is strong precedent for it. Preceding studies revealed that loss of lymphatic integrity due to altered signaling in VEGFR3 within lymphatic lacteals leads to increased fecal fat load^22,23^. Furthermore, conditions that lead to the closure of intercellular junctions in the lacteals also prevents dietary fat uptake and diverts it to the feces^24^.

The causal root of lymphatic changes after small bowel resection is likely multifactorial, with chronic disturbance of flow downstream of the surgery likely having a major effect on lymphatic phenotype and likely resulting in prolonged inflammation. However, the mechanisms of impaired lymph transport remain unknown. After massive intestinal resection, there is a critical, programmed compensatory response in the epithelium resulting in both adaptation (with increased enterocyte proliferation) and apoptosis^25^. Indeed, an emerging concept is that intestinal epithelial cells and the underlying lymphatic vessels coordinately communicate^26,27^. While it is demonstrated that the status of the lymphatic vasculature affects the integrity of intestinal epithelial cells, whether a major change in the status of the epithelium affects the lymphatic vasculature has not been studied. Perhaps future manipulations to improve the status of epithelial cells may improve the lymphatic vasculature or vice versa.

Overall, we have shown that the early lymphatic remodeling structural changes occur chronically after SBR and contribute to functionally compromised lipid absorption. Further studies are necessary to determine if other lymphatic cargo, such as vitamins and immune cells, are also affected after resection. Perturbations in lymphatic function after SBR likely also contribute to greater delivery of luminal endotoxin into the portal vein, thereby contributing to liver injury^28^. Indeed, we have found severe liver injury and fibrosis in resected mice at 15 weeks following SBR when placed on a higher fat diet^29^. Liver injury and fibrosis are the most lethal clinical consequences of SBS^30^. Manipulations of enteral fat may therefore be a critical therapeutic strategy to prevent this significant morbidity.

These data indicate that intestinal lymphatic vessels significantly remodel in the setting of chronic short bowel syndrome. This remodeling results in impaired intestinal transport of fat via the compromised lymphatic architecture, contributing to decreased fatty acid uptake. We believe that these changes may contribute to the development of IFALD, a major morbidity in patients with SBS.

## Materials and methods

### Animals

As described previously, C57BL/6 J 9–12 week-old male mice were obtained from Jackson Laboratories (Bar Harbor, ME)^15^. Prox1-Cre-ER^T2^ (Jax # 022075; originally generated by Srinivasan and Oliver^31^) crossed with Rosa26-tdTomato^fl/fl^ reporter (Jax # 007905) 15–20 week male and female mice were orally treated with 20 mg/ml tamoxifen (Sigma, St. Louis, MO) dissolved in corn oil (50 µg/per gram body weight) every other day over two weeks, inducing Cre recombinase activity and downstream tdTomato expression. Mice were housed in a temperature controlled, specific pathogen-free unit on a 12-h light–dark cycle. All mice were fed a liquid diet (PMI Micro-Stabilized Rodent Liquid Diet LD 101; TestDiet, St. Louis, MO) and water ad libitum. This study was approved by the Washington University in St. Louis Animal Studies Committee (Protocol 20170252 and 20170154) in accordance with the National Institute of Health laboratory animal care and use guidelines. This study is reported in accordance to the ARRIVE guidelines^32^.

### Operations and harvest

Mice underwent either a sham control operation, 50% proximal SBR, or a 75% proximal SBR, as previously described^33,34^. There was also a cohort of mice that did not undergo an operation (non-op), but were treated identically. Mice were placed on liquid diet for 12–24 h preoperatively to minimize the risk of anastomotic obstruction. In brief, for 50% and 75% SBRs, bowel was first exteriorized via a midline laparotomy and then transected 1 to 2 cm distal to the ligament of Treitz and 12 cm or 6 cm proximal to the ileocecal junction, respectively. An end-to-end anastomosis was handsewn with interrupted 9–0 nylon sutures. Sham operations involved a transection with re-anastomosis 12 cm distal to the ileocecal junction. Intraoperative (IO) resected distal bowel was fixed in 10% formalin and embedded in paraffin for immunohistochemistry and immunofluorescence. Postoperatively, mice were kept in an incubator and fasted for 12–24 h before resuming the liquid diet.

### RNA extraction and quantitative reverse transcription-polymerase chain reaction

As described previously, total RNA from proximal and distal intestinal tissue from C57BL/6 J mice harvested approximately 1.5 years postoperatively was isolated using RNeasy Mini kits per the manufacturer’s protocol (Qiagen, Germantown, MD). Proximal intestine was approximately 6 cm harvested from 2 cm past the gastroduodenal junction and distal intestine was the 6 cm leading up to the ileocecal junction, so as to consistently take the same bowel from all operative types (non-operative, sham, and SBRs)^29^. qRT-PCR was conducted using the ABI StepOnePlus Real-Time PCR system with the *ApoB* primer (forward AAACATGCAGAGCTACTTTGGAG, reverse TTTAGGATCACTTCCTGGTCAAA), *FABP2* primer (forward GGTATGGGACAGGCCTTGCT, reverse GGGCATTGTGGTATAGATGACATC), *MTTP* primer (forward ATGATCCTCTTGGCAGTGCTT, reverse TGAGAGGCCAGTTGTGTGAC), *FABP6* primer (forward AGAAGTTCAAGGCTACCGTGAAGA, reverse CCTCCGAAGTCTGGTGATAGTTG), *CD36* primer (forward GAGAACTGTTATGGGGCTAT, reverse TTCAACTGGAGAGGCAAAGG), and *Abca1* primer (forward GAAGAGAGCATGTGGAGTTCTT, reverse TACTTTACCAGGCCCAGTTTG; all are from Applied Biosystems, Waltham, MA). The relative mRNA levels were estimated from the Eq. (2)^–Δ*Ct*^ (Δ*Ct* = *Ct* of target gene minus *Ct* of 18S rRNA). Fold changes in the mRNA level of genes were calculated with a control group level set at 1. Only transporters with significant fold changes were reported.

### Serum free fatty acid, cholesterol, lathesterol, and campesterol levels

Serum free fatty acid, and cholesterol levels were measured using a commercially available kit (Wako Chemicals, Richmond, VA) on C57BL/6 J at 12–15 weeks after SBR. Serum lathesterol and campesterol levels were measured on C57BL/6 J mice 12–13 weeks after SBR. The lathosterol and campesterol esters in mouse plasma were hydrolyzed with potassium hydroxide, and total lathosterol and campesterol were extracted with liquid–liquid extraction in the presence of d7-lathosterol and d6-campesterol as internal standards for lathosterol and campesterol, respectively. The plasma samples and 8-point calibration samples were derivatized with nicotinic acid to improve the mass spectrometric sensitivity. The lathosterol and campesterol were separated by reversed phase liquid chromatography and detected with positive multiple-reaction monitoring on an Applied Biosystems Sciex 4000QTRAP tandem mass spectrometer. The peak integration and construction of standard curve were performed with Analyst 1.6.3. The calibration curve was constructed by plotting the peak area ratios of analyte to internal standard versus the corresponding concentrations using 1/x^2^ weighted least square regression. Serum free fatty acid, and cholesterol levels were performed on mice with confirmed adaptation; adaptation was not assessed in mice tested for serum campesterol and lathosterol levels.

### Functional cholesterol transport

Intestinal uptake of cholesterol was measured using TopFluor-cholesterol (Avanti Polar Lipids, Alabaster, AL) in C57BL/6 J mice at post-operative week 31^35,36^. Mice were fasted for six hours during the mouse dark cycle and then gavaged with TopFluor (8 μg/gm body weight) with methyl-β-cyclodextrin (1 mg/gm body weight; Sigma-Aldrich, St. Louis, MO). Tail vein serum was measured at time points 0, 1, 2, 4, and 6 h after gavage. Methyl-β-cyclodextrin is given with TopFluor in order to deplete already present plasma membrane cholesterol^37,38^. Serum fluorescence was determined using Cytation 5 multiplate reader (Biotek, Winooski, VT). Mice were excluded if fluorescence curve was not achieved secondary to a technical gavage error resulting in post-gavage emesis and incomplete distribution of gavage bolus.

### Food consumption and fecal fat content

Food consumption and fecal fat content were analyzed on mice aged approximately one year after operation. After acclimation to individual metabolic cages, food intake was measured and feces were collected for two days. As previously described, fecal fat content was determined gravimetrically^39^. In brief, dried feces (0.2gm) was solubilized overnight in 1.6 mL water and extracted in 5 mL chloroform:methanol (2:1). The organic phase was transferred into a pre-weighed vial and the homogenate was re-extracted again with 2 mL of cholorform:methanol (2:1). The organic phase was transferred again and, in total, was dried under nitrogen and reweighed to determine the lipid mass. Percent fat absorption was determined by normalizing the lipid mass to food consumption.

### Measurement of body composition

Accretion of body fat mass and lean mass were measured approximately one year after operation using a quantitative nuclear magnetic resonance instrument (Echo Medical Systems, Houston, TX)^40^.

### Functional chylomicron transport

Triglyceride transport was measured in C57BL/6 J mice at approximately one-year postoperatively. Mice were fasted for six hours overnight and then gavaged with C16-Bodipy labeled olive oil (10 μg/gm body weight; Invitrogen, Carlsbad, CA). Tail vein serum was measured at time points 0, 1, 2, 4, and 6 h after gavage. Serum fluorescence was determined using Cytation 5 multiplate reader (Biotek, Winooski, VT). Mice were excluded if fluorescence curve was not achieved secondary to a technical gavage error resulting in post-gavage emesis and incomplete distribution of gavage bolus.

### Bile acid absorption

C57BL/6 J mice approximately 1 year after operation were fasted for six hours overnight and then gavaged with 20% intralipid (7.5μL/gm body weight; Sigma-Aldrich; St. Louis, MO). Tail vein serum was measured at time points 0, 30, 60, and 120 min after gavage. Serum bile acids were measured using a commercially available kit (CrystalChem; Elk Grove Village, IL). Mice were excluded if curve was not achieved secondary to a technical gavage error resulting in post-gavage emesis and incomplete distribution of gavage bolus.

### Immunohistochemistry and immunofluorescence

As described previously, intestinal tissue from 50% SBR and sham control mice 1 cm distal to their anastomosis was harvested 12 weeks post-operatively, fixed in 10% formalin and embedded in paraffin similar to the IO samples taken from the distal bowel resected in the 50% SBR mice at operation^15^. Additionally, proximal gut was obtained from both sham and 50% SBR mice 15 weeks after operation at approximately 5 cm from the gastro-duodenal junction. Longitudinal, 5-μm thick sections were created and hematoxylin and eosin stained for villus height measurement to asses for structural adaptation (NIS elements AR 4; Nikon, Melville, NY), which was set at > 15% increase from IO samples^41^. For immunofluorescence, slides were deparaffinized and antigen retrieval (Diva Decloaking solution, Biocare Medical, Concord, CA) was performed under pressure for 10 min. Slides were blocked in donkey serum (5%, Sigma-Aldrich), bovine serum albumin (1%, Sigma-Aldrich), and Triton-X100 (0.03%, Pharmacia Biotech). Slides were incubated overnight at 4 °C with primary antibodies diluted in 0.2% bovine serum albumin and then secondary antibodies were added for 1 h. Antibodies used include rabbit anti-mouse Lymphatic vessel endothelial hyaluronic acid receptor (LYVE-1; 1:600, Abcam, ab14917), Cy3-conjugated donkey anti-rabbit IgG (1:400, Jackson ImmunoResearch, 711-165-152) and FITC-conjugated mouse anti-mouse alpha-smooth muscle actin IgG (1:500, Sigma, F3777, Clone #1A4). A confocal microscope (Leica SPE and SP8) was used to capture images and blinded analysis was performed using Imaris (Bitplane, Switzerland) and FIJI (ImageJ) software (National Institute of Health, Bethesda, MD) of at least 6 mm of intestine for each sample. LYVE-1^+^, which is a marker of lymphatic endothelial cells, vessel structures present in the lamina propria were defined as mucosal lymphatics and slides were excluded if staining was of poor quality^42^.

### Whole mount mesenteric imaging

Mesentery from Prox1-Cre-ER^T2^xRosa26^LSL^tdTomato mice who were both post-operative week 13 from 50% SBR and sham control operations and also postoperative 1.5 years from 75% SBR and sham control operations was pinned and fixed in 4% paraformaldehyde + 30% sucrose for at least 12 h and then transferred into phosphate-buffered saline, as described previously^15^. The expression of Prospero homeobox protein 1 (PROX1), which governs lymphangiogenesis, is restricted to lymphatic endothelial cells^43^. Fluorescent imaging of the lymphatic branches distal to the anastomosis was then performed (Leica M205FA). Blinded image analysis, including average branch width and area of budding structures was performed using Imaris (Bitplane, Switzerland) and FIJI (ImageJ) software (National Institute of Health, USA) on the postoperative week 13 50% SBR and sham samples.

### Functional lymphatic flow

In mice 12–14 weeks after SBR, anesthetized mice were placed in a custom-built stage to perform intravital recordings using our fluorescence stereoscope, as described previously^44^. Physiological conditions were maintained by a temperature-controlled metal stage with 3 temperature sensors (Dual Channel Temperature Controller, Warner Instruments, TC-344C), controlling the temperature independently at 3 sites at ~ 36–37 °C. Intestine and mesentery were placed over a SYLGARD^®^ 184 (Dow Corning, Nidland, MI) stage and securely attached with fine pins (Fine Science Tools, 26002-20). A midline incision of the peritoneum was used to expose the cecum and terminal ileum, along with the mesentery and mesLNs. Mesentery and intestine were kept moist and under controlled temperature with buffer using a peristaltic pump and In-Line Solution Heater (Harvard Apparatus, 64-0102). To address the intestinal lymphatic draining function, we injected ~ 1.5 µL of 2000 kDa FITC-Dextran (Sigma-Aldrich, FD2000S, or Invitrogen, D7137) into a Peyer’s patch in the ileum, which was the closest to the anastomosis able to be reached in sham and resected mice and the third from the cecum in non-operative mice. After, serum fluorescence, obtained via tail vein, was determined using Cytation 5 multiplate reader (Biotek, Winooski, VT) at 0, 5, 10, 15, 30, 45, and 60 min after the Peyer’s patch injection and presented as fold change from baseline.

### Statistical analysis

Statistical analysis was performed GraphPad-Prism 6 software (La Jolla, CA). Levels of triglyceride, free fatty acid, cholesterol, campesterol, lathosterol, TopFluor cholesterol, C16-Bodipy, fecal fat, mRNA expression levels, and bile acid levels, were analyzed using a one-way ANOVA with Tukey’s multiple comparisons between groups. Intestinal adaptation (increases in villus height) and lymphatic network characteristics were analyzed using the unpaired Student’s t test. A *p* value of < 0.05 was considered significant. Data are expressed as the mean + / − SEM with significance of multiple comparison analyses displayed.

## Abbreviations

SBS: Short bowel syndrome
SBR: Small bowel resection
Non-op: Non-operative
IO: Intraoperative
POD: Post-operative day
